# Relationship between vascular endothelial growth factor -2578C > a gene polymorphism and lung cancer risk: a meta-analysis

**DOI:** 10.1186/s12881-019-0938-0

**Published:** 2020-01-29

**Authors:** Hui-liu Zhao, Jia-hua Yu, Ling-sha Huang, Pei-zhang Li, Ming Lao, Bo Zhu, Chao Ou

**Affiliations:** Department of Clinical Laboratory Medicine, The Affiliated Tumor Hospital of Guangxi Medical University, Nanning, Guangxi Province 530021 People’s Republic of China

**Keywords:** Lung cancer, Vascular endothelial growth factor (VEGF), -2578C > A, Gene polymorphism, Meta-analysis

## Abstract

**Background:**

Several reports were published on the relationship between the vascular endothelial growth factor (VEGF) -2578C > A gene polymorphism and lung cancer risk; however, the results are debatable. This meta-analysis was conducted to assess the relationship between VEGF -2578C > A gene polymorphism and lung cancer risk.

**Methods:**

The associated literatures were identified on the 1st of September 2018 from CBM-disc (China Biological Medicine Database) and PubMed.

**Result:**

A total of 14 reports were recruited into our meta-analysis to assess the association between VEGF -2578C > A gene polymorphism and lung cancer susceptibility. There was a marked association between VEGF -2578C > A A allele / CC genotype and lung cancer risk in overall and Asian populations (overall populations: A allele: OR = 1.26, 95% CI: 1.08–1.46, *P* = 0.003; CC genotype: OR = 0.72, 95% CI: 0.54–0.95, *P* = 0.02; Asians: A allele: OR = 1.33, 95% CI: 1.15–1.55, *P* = 0.0002; CC genotype: OR = 0.68, 95% CI: 0.50–0.93, *P* = 0.01). However, VEGF -2578C > A gene polymorphism was not associated with the risk of lung cancer in Caucasians.

**Conclusion:**

VEGF -2578C > A A allele / CC genotype is associated with the lung cancer susceptibility in Asians and in overall populations.

## Background

Lung cancer is a cancer with less than 15% survival rate and is a leading cause of patients’ death worldwid e[1–4]. It is a complex process requiring the acquisition of genetic mutations which confer the malignant phenotype as well as epigenetic alteration s[5]. Unfortunately, the number of lung cancer related deaths is rapidly increasing each year, and the early diagnosis is crucial to increase the curability chance of patients.

Some genes were found to be associated with the risk of lung cance r[6–8]. The vascular endothelial growth factor (VEGF), is one of the key growth factors, that regulates vascular development and angiogenesis and plays an important role in the growth and progression of human cancers, including lung carcinom a [9, 10]. The current evidence indicated that VEGF gene polymorphism is associated with the susceptibility of some cancer s[11]. There is lack of good diagnostic methods that predict the risk of lung cancer, and which etiology is complicated and not clear.

Several reports were published on the relationship between VEGF -2578C > A gene polymorphism and lung cancer susceptibility. We conducted this meta-analysis to evaluate the association between VEGF -2578C > A gene polymorphism and the risk of lung cancer.

## Methods

### Search strategy

The relevant literature was searched and included using the electronic databases of CBM-disc (China Biological Medicine Database) and PubMed on September the 1st, 2018. The retrieval strategy of “(vascular endothelial growth factor OR VEGF) AND (lung cancer OR lung carcinoma) AND (polymorphism OR polymorphisms)” was entered into the databases. The additional literature was obtained from cited references in recruited studies.

### Inclusion and exclusion criteria

#### Inclusion criteria

(1) Patients outcomes must be lung carcinoma; (2) There should be two comparison groups (lung cancer group vs control group); (3) The studies should show data for VEGF -2578C > A genotype distribution.

#### Exclusion criteria

(1) Editorials and review articles; (2) Case reports; (3) Preliminary outcome not on VEGF -2578C > A gene polymorphism or lung cancer; (4) Studying the role VEGF gene expression in cancers; (5) Multiple publications.

### Data extraction and synthesis

The data was searched and extracted by two investigators that were independent from each eligible study. The investigators analyzed the data based on the first author’s surname, location of the performed study, year of publication, the number of cases and controls for VEGF -2578C > A genotypes and the control source of the control group. Disagreements were resolved by discussion.

### Statistical analysis

Revman 5 (Cochrane Library, UK) was applied to calculate the data extracted from each literature. When the *P* value for the heterogeneity test was less than 0.1, a random effects model was applied. Otherwise, the pooled statistic was counted using the fixed effects model. Odds ratios (OR) were used to express the dichotomous data, and 95% confidence intervals (CI) were also counted. A P value of less than 0.05 was regarded as the pooled OR, to be notably significant. *I*^*2*^ was applied to detect the heterogeneity among the included investigations. According to the source of the controls, sensitivity analysis was also performed (population-based vs hospital-based). Stata 11.0 was used to test the publication bias. The Begg tes t[12] and the Egger tes t[13] were applied to assess the publication bias (*P* < 0.1 was considered significant), when the sample size of included studies was more than ten.

## Results

### Study characteristics

**Fig. 1:**
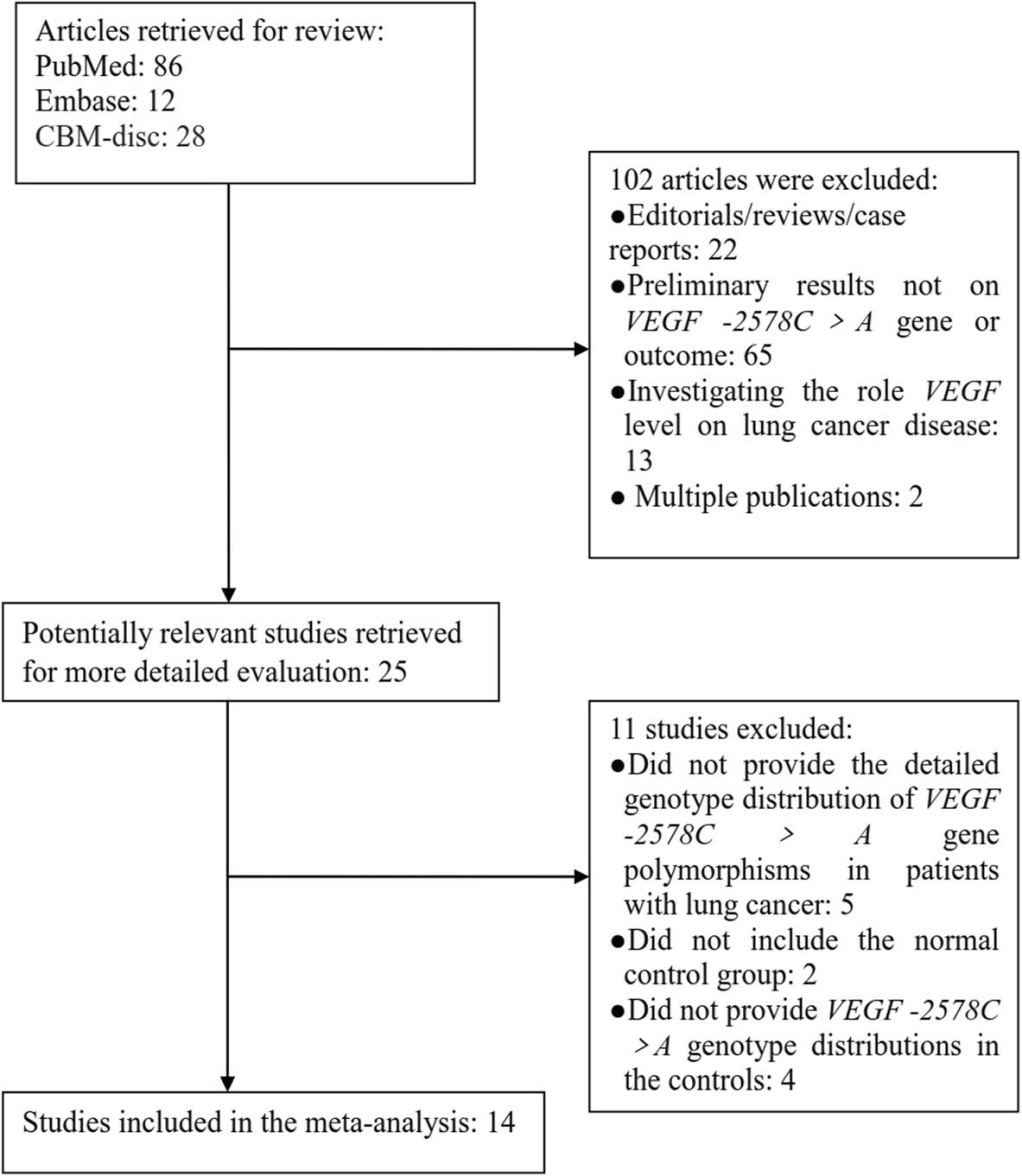
Flow chart of the study search and selection

A total of 14 studie s[14–27] on the association between VEGF -2578C > A gene polymorphism and the susceptibility of lung cancer, were included into this meta-analysis (Table 1 and Fig. 1). The data of our interest were extracted (Table 1). Those 14 investigations contained 3120 patients with lung cancer and 3540 controls. The method for the detection of VEGF -2578C > A gene polymorphism in all the included studies, involved the use of restriction fragment length polymorphism polymerase chain reaction (RFLP-PCR). We have calculated the allele frequencies of the variant allele (A allele) and found that the frequency of A allele in the lung cancer group was 27.7%, and it in control group was 24.2%.

**Table 1:** General characteristics of the included studies in this meta-analysis for VEGF -2578C > A gene polymorphism with lung cancer risk

First author,	Country	Ethnicity	Control	Detecting		Case				Control		
year	/District		source	methods	AA	CA	CC	Total	AA	CA	CC	Total
Wang 2008	China	Asian	Population-base	RFLP-PCR	23	127	227	377	22	107	287	416
Liang 2009	China	Asian	Population-base	RFLP-PCR	14	28	129	171	4	56	112	172
Wang 2009	China	Asian	Population-base	RFLP-PCR	21	104	175	300	16	75	209	300
Liu 2010	China	Asian	Population-base	RFLP-PCR	13	74	85	172	7	65	112	184
Li 2010	China	Asian	Population-base	RFLP-PCR	12	25	113	150	3	49	98	150
Yuan 2011	China	Asian	Population-base	RFLP-PCR	18	108	125	251	8	93	154	255
Li 2012	China	Asian	Population-base	RFLP-PCR	12	35	108	155	3	33	114	150
de Mello 2013	Portugal	Caucasian	Population-base	RFLP-PCR	26	75	43	144	27	73	44	144
Geng 2013	China	Asian	Population-base	RFLP-PCR	16	118	126	260	9	89	162	260
Wang 2014	China	Asian	Population-base	RFLP-PCR	21	104	175	300	16	75	209	300
Deng 2014	China	Asian	Population-base	RFLP-PCR	6	33	26	65	7	41	62	110
Liu 2015	China	Asian	Population-base	RFLP-PCR	20	164	230	414	23	138	177	338
Krupnova 2015	Belarus	Caucasian	Hospital-based	RFLP-PCR	31	90	41	162	91	186	83	360
Naykoo 2017	India	Asian	Population-base	RFLP-PCR	5	170	24	199	55	145	201	401

*RFLP-PCR* Restriction fragment length polymorphism polymerase chain reaction, *VEGF* Vascular endothelial growth factor

### Relationship between VEGF -2578C > a gene polymorphism and lung cancer susceptibility in overall populations

**Fig. 2:**
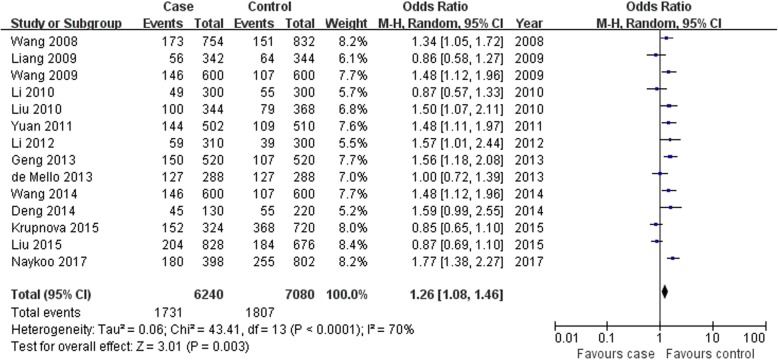
Association between VEGF -2578C > A A allele and lung cancer susceptibility in overall populations

**Fig. 3:**
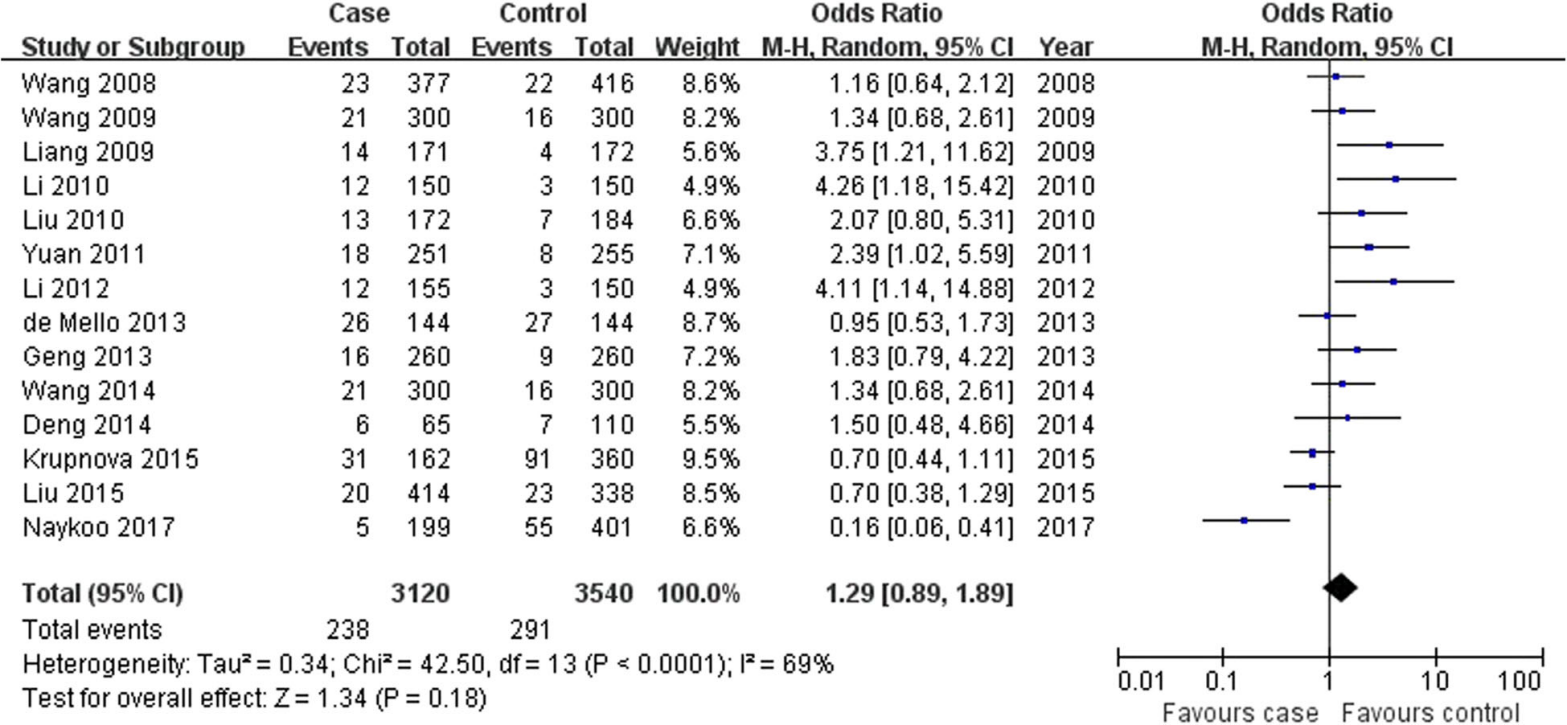
Association between VEGF -2578C > A AA genotype and lung cancer susceptibility in overall populations

**Fig. 4:**
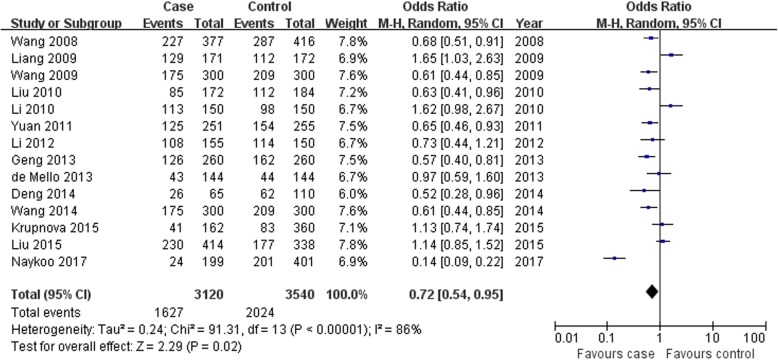
Association between VEGF -2578C > A CC genotype and lung cancer susceptibility in overall populations

VEGF -2578C > A A allele and CC genotype were associated with lung cancer risk; however, the AA genotype was not found in overall populations (A allele: OR = 1.26, 95% CI: 1.08–1.46, *P* = 0.003, Fig. 2; AA genotype: OR = 1.29, 95% CI: 0.89–1.89, *P* = 0.18, Fig. 3; CC genotype: OR = 0.72, 95% CI: 0.54–0.95, *P* = 0.02, Fig. 4; Table 2).

**Table 2:** Meta-analysis of the association of VEGF -2578C > A gene polymorphism with lung cancer risk

Genetic contrasts	Group and subgroups	Studies number	Q test P-value	Model selected	OR (95%CI)	P
A vs C	Overall	14	<0.0001	Random	1.26 (1.08,1.46)	0.003
	Asian	12	0.001	Random	1.33 (1.15,1.55)	0.0002
	Caucasian	2	0.43	Fixed	0.90 (0.74,1.11)	0.33
AA vs CA + CC	Overall	14	<0.0001	Random	1.29 (0.89,1.89)	0.18
	Asian	12	0.0001	Random	1.45 (0.92,2.28)	0.11
	Caucasian	2	0.42	Fixed	0.78 (0.55,1.12)	0.18
CC vs CA + AA	Overall	14	<0.00001	Random	0.72 (0.54,0.95)	0.02
	Asian	12	<0.00001	Random	0.68 (0.50,0.93)	0.01
	Caucasian	2	0.64	Fixed	1.06 (0.76,1.47)	0.74
Sensitivity analysis according to source of the controls from population-based						
A vs C	Overall	13	0.0008	Random	1.30 (1.05,1.72)	0.0003
AA vs CA + CC	Overall	13	0.0002	Random	1.39 (0.92,2.08)	0.12
CC vs CA + AA	Overall	13	<0.00001	Random	0.70 (0.52,0.93)	0.02
Sensitivity analysis according to source of the controls from hospital-based						
T vs C	Overall	1	–	Fixed	0.85 (0.65,1.10)	0.21
TT vs CT + CC	Overall	1	–	Fixed	0.70 (0.44,1.11)	0.13
CC vs CT + TT	Overall	1	–	Fixed	1.13 (0.74,1.74)	0.58

### Relationship between VEGF -2578C > a gene polymorphism and lung cancer susceptibility in Asians

In this meta-analysis, VEGF -2578C > A A allele, and CC genotype were associated with the risk of lung cancer in Asians; however, the AA genotype was not (A allele: OR = 1.33, 95% CI: 1.15–1.55, *P* = 0.0002; AA genotype: OR = 1.45, 95% CI: 0.92–2.28, *P* = 0.11; CC genotype: OR = 0.68, 95% CI: 0.50–0.93, *P* = 0.01; Table 2).

### Relationship between VEGF -2578C > a gene polymorphism and lung cancer susceptibility in Caucasians

In this meta-analysis, VEGF -2578C > A gene polymorphism was not associated with the susceptibility of lung cancer in Caucasians (A allele: OR = 0.90, 95% CI: 0.74–1.11, *P* = 0.33; AA genotype: OR = 0.78, 95% CI: 0.55–1.12, *P* = 0.18; CC genotype: OR = 1.06, 95% CI: 0.76–1.47, *P* = 0.74; Table 2).

### Sensitivity analysis

The sensitivity analysis for the association between VEGF -2578C > A gene polymorphism and lung cancer susceptibility was also performed by the source of the controls (population-based vs hospital-based). In the sensitivity analysis using population-based, the VEGF -2578C > A A allele and CC genotype were associated with lung cancer risk; however, the AA genotype was not (Table 2). In the sensitivity analysis using the hospital-based control, VEGF -2578C > A gene polymorphism was not associated with lung cancer susceptibility (Table 2).

### Evaluation of publication bias

**Fig. 5:**
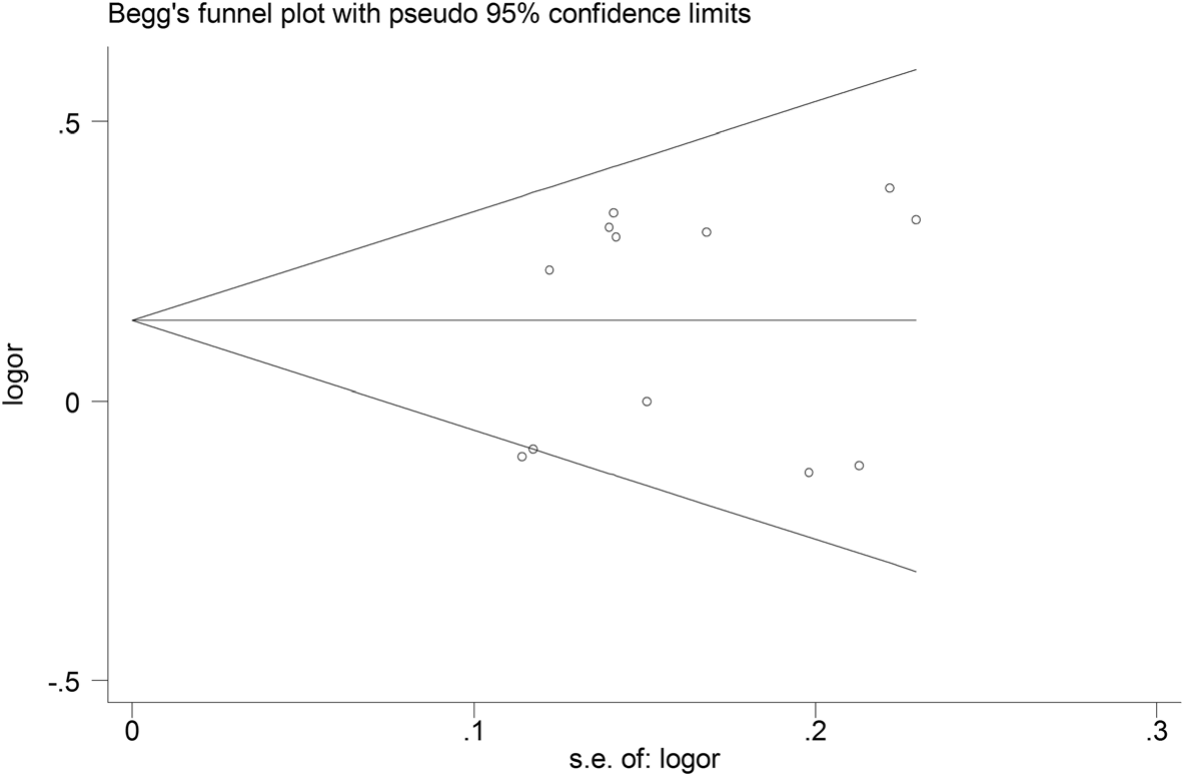
Funnel plot to assess publication bias for the association of VEGF -2578C > A gene polymorphism with lung cancer susceptibility in overall populations

No publication bias was found for the overall populations (Begg *P* = 0.807, funnel plot was presented in Fig. 5; Egger *P* = 0.505), and Asians (Begg *P* = 0.938, Egger *P* = 0.827).

## Discussion

VEGF is regarded as an important factor taking part in the inactivation of pro-carcinogens, which contribute to cancer. In this study, we included 14 studies into our meta-analysis. We investigated whether the VEGF -2578C > A gene polymorphism is a valuable indicator for lung cancer susceptibility, and attempted to draw robust results. In our meta-analysis, we found that there was a marked association between VEGF -2578C > A A allele / CC genotype and lung cancer risk in overall and Asian populations. However, VEGF -2578C > A gene polymorphism was not associated with the risk of lung cancer in Caucasians. The sample size of the included studies was larger than that of other meta-analyses, and the results on the association between VEGF -2578C > A gene polymorphism and lung cancer risk might be more robust. The pooled OR for A allele was 1.26 for overall populations and 1.33 for Asians; while, for the CC genotype the odds ratio was less than 1. It indicated that high CC genotype was a protective genotype (good genotype); however, A allele was a negative gene allele. The AA genotype was not associated with lung cancer risk, which might due to the small sample size of included studies, and therefore, more studies should be conducted to confirm this result. Nevertheless, the results for the Caucasian population were less robust, also due to the small number of included studies. Additional studies should be performed to confirm this result.

In the sensitivity analysis by the controls source, we found that, in the sensitivity analysis using the population-based control, the VEGF -2578C > A A allele / CC genotype was associated with lung cancer susceptibility. However, in the sensitivity analysis using the hospital-based control, VEGF -2578C > A gene polymorphism was not associated with lung cancer susceptibility.

Publication bias was also analyzed, and we found that there was no publication bias for overall and Asians populations. This suggests that the conclusion from our meta-analysis was robust. However, additional well-designed studies should be performed to confirm this result in the future.

In a previous study, Deng et al .[15] recruited four reports into their study using a meta-analysis method, and showed that the CC genotype was associated with lung cancer; however, A allele and AA genotype were not. Chen et al .[28] also included four studies into their meta-analysis, and obtained a similar result. Lin et al .[29] included seven studies into their meta-analysis, and reported that the A allele was associated with lung cancer, but the AA and CC genotypes were not. Our meta-analysis indicated that there was an association between A allele, CC genotype and lung cancer risk in overall and Asian populations. The sample size in our meta-analysis was larger than the previous meta-analyses, and the outcome in our meta-analysis might be more robust. In this study, we found that the CC genotype is the dominant genotype associated with lung cancer risk. We speculated that the CC genotype might be associated with high levels of VEGF, and that the increased VEGF was associated with lung cancer risk.

In the test of publication bias, two points were located on or out of scope, indicating publication bias. We deleted the two studies and conducted a further meta-analysis, and found the results were similar.

## Conclusions

The results in our study support that VEGF -2578C > A A allele / CC genotype was associated with lung cancer susceptibility in overall and Asian populations. However, additional well-designed investigations of this association are required to confirm these results.

## Data Availability

The datasets used and/or analyzed during the current study are available from the corresponding author on reasonable request.

## Abbreviations

VEGF: Vascular endothelial growth factor
